# Transcriptomic Analysis Reveals Involvement of the Macrophage Migration Inhibitory Factor Gene Network in Duchenne Muscular Dystrophy

**DOI:** 10.3390/genes10110939

**Published:** 2019-11-18

**Authors:** Salvo Danilo Lombardo, Emanuela Mazzon, Katia Mangano, Maria Sofia Basile, Eugenio Cavalli, Santa Mammana, Paolo Fagone, Ferdinando Nicoletti, Maria Cristina Petralia

**Affiliations:** 1 Department of Biomedical and Biotechnological Sciences, University of Catania, 95123 Catania, Italy; salvo.lombardo.sdl@gmail.com (S.D.L.); kmangano@unict.it (K.M.); sofiabasile@hotmail.it (M.S.B.); ferdinic@unict.it (F.N.); 2 IRCCS Centro Neurolesi Bonino Pulejo, C.da Casazza, 98124 Messina, Italy; emanuela.mazzon@irccsme.it (E.M.); santa84ma@libero.it (S.M.); m.cristinapetralia@gmail.com (M.C.P.)

**Keywords:** macrophage migration inhibitory factor, Duchenne muscular dystrophy, dystrophic muscle diseases

## Abstract

Duchenne muscular dystrophy (DMD) is a progressive hereditary muscular disease with X-linked recessive inheritance, that leads patients to premature death. The loss of dystrophin determines membrane instability, causing cell damage and inflammatory response. Macrophage migration inhibitory factor (MIF) is a cytokine that exerts pleiotropic properties and is implicated in the pathogenesis of a variety of diseases. Recently, converging data from independent studies have pointed to a possible role of MIF in dystrophic muscle disorders, including DMD. In the present study, we have investigated the modulation of MIF and MIF-related genes in degenerative muscle disorders, by making use of publicly available whole-genome expression datasets. We show here a significant enrichment of MIF and related genes in muscle samples from DMD patients, as well as from patients suffering from Becker’s disease and limb-girdle muscular dystrophy type 2B. On the other hand, transcriptomic analysis of in vitro differentiated myotubes from healthy controls and DMD patients revealed no significant alteration in the expression levels of MIF-related genes. Finally, by analyzing DMD samples as a time series, we show that the modulation of the genes belonging to the MIF network is an early event in the DMD muscle and does not change with the increasing age of the patients, Overall, our analysis suggests that MIF may play a role in vivo during muscle degeneration, likely promoting inflammation and local microenvironment reaction.

## 1. Introduction

Duchenne muscular dystrophy (DMD) is a progressive hereditary muscular disease with X-linked recessive inheritance, with an incidence ranging from 10.71 to 27.78 live-born males per year per 100,000 [1], and worldwide estimated prevalence of 4.78 per 100,000 [1]. The natural progression of the disease includes hypertrophy of cardiac muscle and diaphragmatic contraction impairment, that leads patients to premature death [2]. The pathogenesis of DMD depends on the absence or altered forms of the dystrophin protein. This protein is essential for muscular growth and function. It acts as a scaffold in the subsarcolemmal space protein complex and binds the actin, bridging the extracellular and intracellular space [3,4]. The loss of dystrophin determines membrane instability, causing cell damage and inflammatory response [5]. Similar to dystrophin, other molecules have been identified to be involved in the maintenance of membrane stability, such as dysferlin, a protein involved in membrane repair processes that, when altered, determines the limb-girdle muscular dystrophy type 2B (LGMD2B), characterized by chronic muscle inflammation and damage [6]. 

Several factors have been proposed to promote the progression of the disease. In particular, it has been shown that the immune system plays an important role in dystrophic muscle disease pathogenesis, sustaining a continuous inflammatory and fibrotic response [7,8,9]. 

The macrophage migration inhibitory factor (MIF) is a proinflammatory cytokine secreted by activated T cells and macrophages that exerts proliferative, chemotactic, and anti-apoptotic functions. Its role has been well established in several pathologies of heterogeneous clinical symptomatology, including inflammatory and autoimmune diseases [10,11,12] and cancer [13,14] (reviewed by [15]). Reimann et al. [16] have observed increased MIF protein levels in dermatomyositis, polymyositis, and sporadic inclusion body myositis muscle samples, suggesting a role for MIF in the regenerative response to muscle fiber damage. In addition, it has been shown that MIF regulates muscle metabolism [17]. These data suggest that MIF may exert a homeostatic and regulatory effect in muscle cells.

MIF mediates its action through CD74 (also known as HLA-DR antigens-associated invariant chain), which recruits the cell-surface glycoprotein CD44, and the co-receptors, CXCR2, CXCR4, and CXCR7, as well as the intracellular protein JAB1 (encoded by the *COPS5* gene), determining the activation of a variety of signaling cascades, including the MAPK, PI3K/AKT, and NF-kB pathways [18].

In the present study, we have investigated the expression of MIF and related gene networks in DMD by making use of publicly available whole-genome expression profiles of human muscle cellular models and bioptic samples.

## 2. Materials and Methods

### 2.1. Network Construction

Genes functionally related to MIF were obtained from the GeneMania database (http://genemania.org/) [19]. GeneMania integrates publicly available genomics and proteomics data, including data from gene and protein expression profiling studies, and molecular interaction pathways, to find related genes [19]. The search was conducted imputing the following terms: *MIF*, *DDT*, *CD74*, *CD44*, *CXCR2*, *CXCR4*, *CXCR7* (a.k.a. *ACKR3*), and *COPS5*. The genes were selected based on Gunther et al. [15]. The parameters were the following: Maximum resultant genes, 40; maximum attributes, 20; query, dependent weighting.

### 2.2. Dataset Selection and Analysis

The NCBI Gene Expression Omnibus (GEO) database (http://www.ncbi.nlm.nih.gov/geo/) was used to identify microarray datasets comparing muscle transcriptomic profiles from healthy donors vs. DMD patients. Two datasets were included in the meta-analysis, GSE38417, and GSE6011. Briefly, the GSE38417 dataset included six samples of skeletal muscle biopsy from healthy people and 16 samples from DMD patients. The GSE6011 dataset included 14 healthy control samples and 23 samples from DMD patients [20]. The web-based application, ImaGEO [21] was used to perform the meta-analysis (http://bioinfo.genyo.es/imageo/) [21], and the gene ontology of the identified Differentially Expressed Genes (DEGs). 

The GSE109178 dataset was used for the evaluation of the involvement of the MIF network in the degenerative muscle disorders, Becker’s muscular dystrophy (BMD), and LGMD2B [22]. The dataset contained whole-genome expression profiles from six healthy donors, 11 BMD patients (bearing abnormal dystrophin protein), and eight LGMD2B patients (bearing mutations in the *DYSF* gene) [22]. The Affymetrix Human Genome U133 Plus 2.0 Array was used for the generation of the dataset [22].

For the comparative evaluation of the MIF network in in vitro differentiated human myotubes, we interrogated the GSE79263 dataset [23]. The dataset comprised gene expression profiles from two healthy and three DMD patients [23]. The Illumina HumanHT-12 V4.0 Expression BeadChip platform was used for the generation of this dataset [23]. Raw data were background corrected followed by quantile normalization.

### 2.3. Statistical Analysis

For the meta-analysis of the GSE38417 and GSE6011 datasets, a fixed-effect model of effect size measure was used to integrate gene expression patterns from the two datasets. Genes with an adjusted *p*-value  <  0.05 and an │effect size│ > 2 were identified as DEGs and selected for further analysis.

Linear Models for Microarray Data (LIMMA) was used to assess statistically significant differences between groups, in the GSE10978 and GSE79263 datasets. Genes with an adjusted *p*-value  <  0.05 and a │fold change│ > 2 were identified as DEGs.

The Bayesian Estimation of Temporal Regulation (BETR) [24] was used to determine the modulation of the genes of interest in patients at different ages. BETR is a linear random-effects modeling statistical method that takes into account the correlations between samples and the time-points. Each gene is given a probability of differential expression that derives from an empirical Bayes approach using the whole data set to reduce the number of parameters to be estimated [24]. For the analysis, patients were arbitrarily grouped into three age-defined classes: Class 1: <2 yrs (*n* = 5), Class 2: 3–4 yrs (*n* = 6), and Class 3: 5–8 yrs (*n* = 5).

Principal component analysis (PCA) was conducted on the genes of interest to assign the general variability in the data to a reduced set of variables, by using the Multiple Experiment Viewer (MeV) software (v. 4.9.0) [25].

For the evaluation of the significance of enrichment of the upregulated and downregulated DEGs among the MIF network genes, a Chi-square test was performed. A *p*-value < 0.05 was considered to be statistically significant.

## 3. Results

### 3.1. Generation of the MIF Network

The GeneMania database was used to construct the MIF network, based on physical interaction, co-expression, predicted, co-localization, pathway, genetic interactions, and shared protein domains. Input genes were: *MIF*, *DDT*, *CD74*, *CD44*, *CXCR2*, *CXCR4*, *CXCR7* (a.k.a. *ACKR3*), and *COPS5*. Overall, the network included 48 unique genes with 81 multi-edge node pairs (Supplementary Table S1).

### 3.2. Meta-Analysis of Gene Expression in Duchenne Muscular Dystrophy

**Figure 1:**
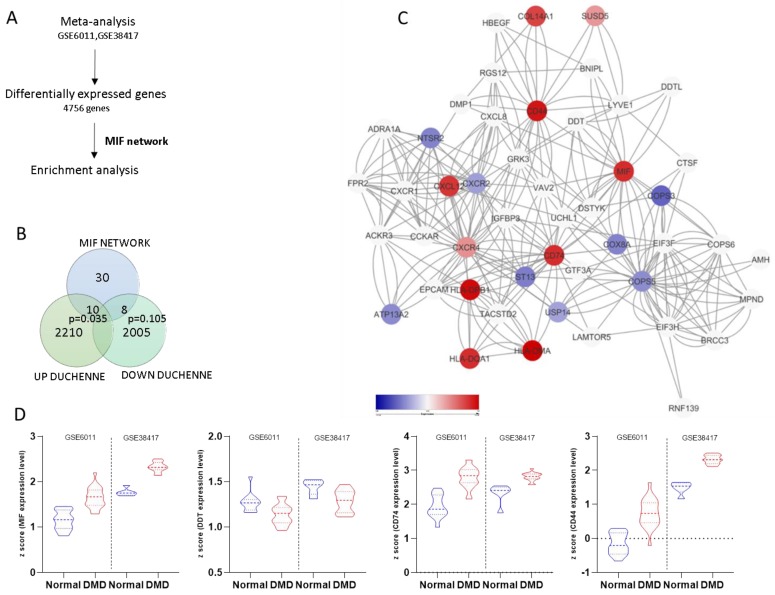
Enrichment of the migration inhibitory factor (MIF) network in Duchenne muscular dystrophy (DMD). Study layout (**A**). Overlapping between the differentially expressed genes (DEGs) in DMD samples, as determined in the meta-analysis of the GSE6011 and GSE38417 datasets, and the MIF network (**B**). MIF network showing the DEGs identified in the meta-analysis. Nodes are color-coded based on the observed Effect Size (**C**). Z score of the expression levels of MIF, DDT, CD74 and CD44 in the GSE6011, and GSE38417 datasets (**D**).

Two GEO datasets were identified for the subsequent evaluation of the involvement of the MIF pathway in DMD (Figure 1A). A total of 20 samples from healthy controls and 39 skeletal muscle biopsies from DMD patients were used in the meta-analysis. We identified 7107 and 1586 differentially expressed genes in the GSE38417 and in the GSE6011 dataset, respectively. The meta-analysis identified 4756 DEGs between healthy and DMD samples. Gene ontology analysis revealed that the top three biological processes enriched by the upregulated DEGs were: “Immune response” (GO:0006955), “immune system response” (GO:0002376) and “defense response” (GO:0006952) (Supplementary Figure S1). Among the upregulated DEGs, Venn diagram analysis identified 10 genes overlapping the MIF network, reaching the statistical significance (*p* = 0.035) (Figure 1B,C). On the other hand, eight out of the 2013 downregulated DEGs overlapped the MIF network, without reaching the statistical significance (Figure 1B,C). Figure 1D shows the expression levels of the four principal hubs (MIF, DDT, CD74, and CD44) of the MIF network in the two individual microarray datasets used for the meta-analysis (Figure 1D). In order to determine whether the involvement of the MIF network was recapitulated in vitro, we interrogated the GSE79263 dataset, which contains the transcriptional profiles of primary myotubes from healthy and DMD patients. As shown in Table 1, no statistically significant differences were observed in the expression levels of the MIF-related genes between healthy and DMD samples (Table 1).

### 3.3. Enrichment of the MIF Network in Becker Disease and Limb-Girdle Muscular Dystrophy Type 2B

**Figure 2:**
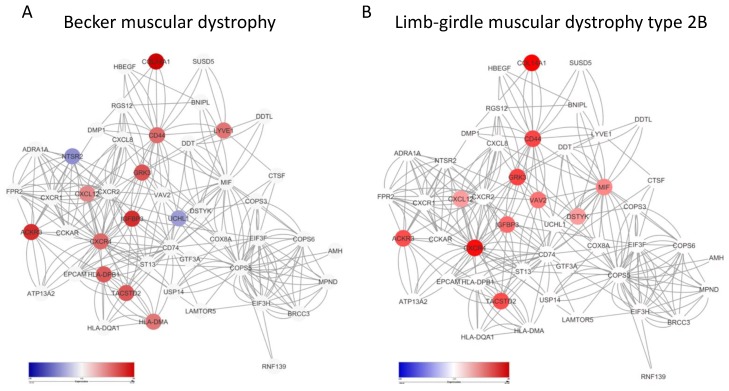
MIF network in dystrophic muscle diseases. MIF network showing DEGs as nodes color-coded based on fold change, in Becker’s disease (**A**) and in limb-girdle muscular dystrophy type 2B (**B**), as determined in the GSE79263 dataset.

In order to evaluate whether the involvement of the MIF network was peculiar to the DMD muscle or was rather a pathogenetic pathway common to other muscular dystrophic disorders, we analyzed the GSE109178 dataset that included whole-genome transcriptomic data from healthy donor muscle samples and biopsies from Becker’s and LGMD2B patients. As shown in Figure 2, in Becker’s disease, 11 upregulated DEGs (*p* < 0.05) and two downregulated DEGs, overlapped with genes belonging to the MIF network (Figure 2A). Similarly, in LGMD2B, 11 upregulated DEGs belonged to the MIF network (*p* < 0.05) (Figure 2B).

### 3.4. Modulation of the MIF Pathway in Muscle Biopsies of DMD Patients at Different Ages

**Figure 3:**
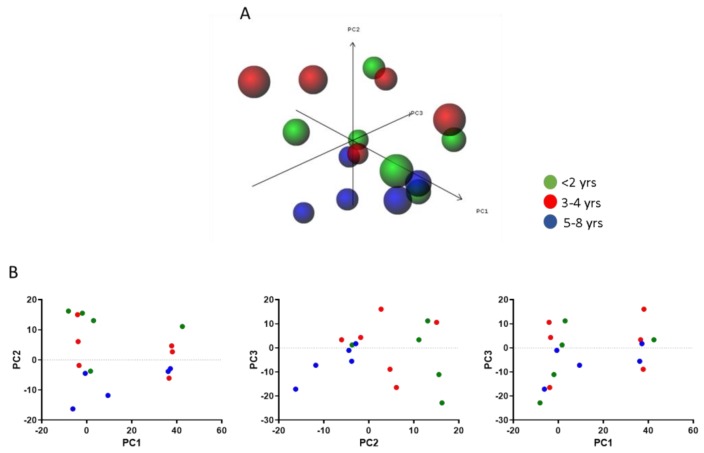
Principal component analysis of MIF network genes in muscle biopsies of DMD patients at different ages, as determined in the GSE38417 dataset. Samples were divided based on DMD patients’ age: <2 yrs (green), 3–4 yrs (red), 5–8 yrs (blue). Data points are projected onto principal components (PC) 1, 2, and 3 (**A**). The same data (**B**), projected onto PC1 and 2, PC2 and 3, and PC1 and 3.

We sought to investigate whether alterations in the expression of MIF-related genes could be observed in the muscles from DMD patients at different ages. To this aim, we used the GSE38417 dataset, which includes data from the muscles of DMD patients who had a broad range of ages. As the disease is usually diagnosed at the age of three to seven years, the first two years are often referred to as presymptomatic. We analyzed the samples as a time series, dividing the samples into three age-defined classes: <2 yrs, 3–4 yrs, 5–8 yrs. BETR analysis revealed that no significant modulation of the MIF-related network occurs at different patients’ age (Table 2). Accordingly, data complexity reduction using PCA for the genes of interest showed that samples do not cluster based on age-related transcriptomic features (Figure 3).

## 4. Discussion

DMD represents an unmet medical need, hence the identification of novel regulatory pathways controlling muscle degeneration, may allow for the development of tailored approaches that delay disease progression, improve the quality of life, and prolong the life of the patients. The use of whole-genome expression data has been extensively used for the identification of novel pathogenic pathways and therapeutic targets in several pathologies including, immunoinflammatory/autoimmune diseases [26,27,28,29,30] and cancer [31,32,33,34,35], and have allowed identification of novel cellular and molecular targets [36,37]. Gene regulatory networks and pathway analysis may help the development of new therapies aimed at increasing muscle protection/regeneration, and some of them have already proven their effectiveness, also in DMD [38]. 

Here, we have first conducted a meta-analysis considering two datasets of muscle biopsies from DMD patients to evaluate the over-representation of the MIF network among the Differentially Expressed Genes. 

We have found that MIF, as well as the receptor CD74, CD44 and CXCR4 are significantly upregulated in DMD. On the contrary, CXCR2 seems to be down-regulated in DMD. This may be relevant in light of the fact that, even if both CXCR4 and CXCR2 converge to CD74 and then CD44 [15,18], CXCR4 shows also a different sub-pathway associated with inflammatory response (NFKB1, NFKBIA, STAT3) and cell survival (AKT1) [18]. From our analysis, CXCL12 was also highly expressed in DMD, and this may have important implications, as the CXCL12/CXCR4 axis is involved in muscle regeneration [5]. Moreover, MIF signaling increases the synthesis of the Tra2α splicing factor, which in turn leads to the transcription of some specific CD44 isoforms, that facilitate extracellular matrix migration and provide binding sites for matrix metalloproteinases (MMPs) and growth factors, such as fibroblast growth factor (FGF) [39]. The MIF receptor, CD74, expressed on the cell surface, recruits CD44, where both proteins become phosphorylated and initiate downstream signal transduction [40]. In monocytes and stromal cells, the initial activation of CD44-associated Src tyrosine kinase and MEK leads to phosphorylation of ERK1/2 MAP kinases, activation of cytosolic phospholipase A2 (cPLA2), and the inhibition of p53 [41]. In B cells, CD44-associated Syk tyrosine kinase leads to Akt phosphorylation and downstream NFκB activation [42]. MIF binding to CD74 also results in the intramembrane cleavage of CD74 involving the positive regulation of B cell maturation [43], the activation of p65-NFκB, upregulation of TAp63, and stimulation of Bcl-2, leading to enhanced cell survival [44]. MIF signaling through the CD74-CXCR4-CXCR7 complex is involved in lymphocyte chemotaxis, particularly in B cells [45]. Since replacement fibrosis contributes to DMD pathology [46,47,48], the present observation of the upregulated expression of MIF is extremely important, in order to fully understand DMD pathogenesis and find possible new future therapeutic targets. It is of interest that small molecule MIF inhibitors have been found to prevent fibrosis in an in vivo model of bleomycin-induced pulmonary fibrosis [49], thus dismantling the fibrogenetic role of MIF.

Interestingly, we have not found any significant difference in myotubes from control patients and DMD patients. We hypothesize that the in vitro culture of muscle cells may not reflect the complexity of the environment that exists in vivo. Overall, our data suggest that MIF may play a role during muscle degeneration, likely promoting inflammation and local microenvironment reaction [16]. This is in line with the observation that the main biological processes enriched by the DEGs identified in DMD are associated with immune responses. Moreover, supporting our hypothesis, our analysis shows that MIF, and its related genes, are modulated also in other muscle diseases, i.e., LGMD2B and Becker’s dystrophy. In addition, we have provided evidence that the MIF-related genes do not undergo modulation in DMD patients over a broad range of ages (i.e., from 1 to 8 yrs), and this further suggests that alteration of MIF network is an early event in the DMD muscle degeneration, which is also maintained over time.

We are aware of the limitations of the current work that include the lack of in vitro and in vivo validation. Nonetheless, this paper is the first demonstration of the upregulated network of MIF in DMD patients and represents the first valuable proof-of-concept (POC) that highlights the potential contribution of this family of cytokines to the pathogenesis of the disease. This finding is also of clear translational relevance as it suggests that the MIF family and its receptors may represent suitable therapeutic targets in DMD.

Currently accepted pharmacological treatment for DMD is corticosteroids, in order to suppress muscle inflammation. However, this treatment has limited efficacy and considerable side effects [2,50]. For this reason, new therapies are studied to reduce the inflammation associated with muscle degeneration. In mdx mice, a well-known model used for the study of DMD, daily treatment with nonsteroidal anti-inflammatory drugs (NSAIDs) reduces macrophage infiltration and necrosis but does not modify the percentage of regenerating myofibers [51]. An effective new therapeutic approach consists of reducing NF-kB activity to prevent or delay the onset of muscle dysfunction [52]. 

Today, different options exist to target MIF for the treatment of human patients: small molecule inhibitors, monoclonal antibodies, and nanobodies, and peptide inhibitors. Interestingly, the FDA approved drug, Ibudilast, has been shown to exert an additional action to its original PDE5 inhibition, that entails inhibitory action of MIF [53]. The anti-MIF activity of Ibudilast has propelled attention for the possible repurposing of this drug in immunoinflammatory pathologies where MIF is thought to be implicated, such as multiple sclerosis [54]. In addition, an anti-MIF mAb has been tested in Phase II trials in patients with cancer (NCT02448810, NCT02540356). Both Ibudilast and the anti-MIF mAb may eventually be immediately available for their evaluation in POC Phase II studies in patients with DMD. In a similar manner, the use of MIF and DDT receptor antagonists could be considered when evaluating tailored MIF-DDT therapies for DMD. The anti-CD74 mAb milantuzumab is currently being studied in phase I/II studies for hematological cancers [55] and could represent a potential pharmacological candidate for MIF-DDT tailored interventions. 

It is also of particular relevance in the context of DMD, given the involvement of the MIF network, is that the biological function of MIF may be inhibited by nitrosylation [56]. The lack of endogenous NO secondary to the loss of dystrophin has been hypothesized to play role in DMD pathogenesis since this gas is a potent regulator of skeletal muscle metabolism, mass, function, and regeneration [57]. NO donors might have a dual and synergistic mode of action in DMD that entails NO donation and MIF inhibition. Along this line of research, we propose that along with NO-NSAID, that have been tested successfully in mice and with some efficacy in the clinical setting [57,58], also other NO-donors including the recently characterized lopinavir-NO and ritonavir-NO [59,60,61,62], that have different pharmacological mode of actions than NO-NSAID, and may have higher efficacy, should deserve particular consideration as double-tailored drugs to be used in the treatment of DMD patients. It is worth noting in this regard, that lopinavir-NO has been shown to exert immunomodulatory properties more potent than its parental compound in vitro, and it has been capable of successfully preventing a model of MIF-dependent immunoinflammatory hepatitis [63,64]. This analysis may set the basis to encourage future clinical trials for anti-MIF drugs in disease like DMD, that despite being considered only a genetic disease, is characterized by in important involvement of inflammation.

## 5. Conclusions

Although DMD is considered a progressive hereditary muscular disease, in recent years, many studies have been focused on the impact of the immune system in the progression and symptomatology of this disease. In this paper, we have shown the important role of MIF in DMD, and in other dystrophic muscle diseases, as well, suggesting the potential use of anti-MIF drugs.

## Figures and Tables

**Table 1:** Differential expression analysis of MIF-related genes in in-vitro differentiated myotubes from healthy donors and DMD patients, as determined in the GSE79263 dataset. Values are approximated to four digits.

Symbol	*p*-Values	Adj-*p*-Values	Log Fold-Change	*t*-Statistic
*Ackr3*	0.7736	0.9997	−1.0993	−0.2921
*Adra1a*	0.9940	0.9997	0.0286	0.0076
*Amh*	0.9782	0.9997	0.1045	0.0277
*Atp13a2*	0.9363	0.9997	−0.3327	−0.0810
*Bnipl*	0.9948	0.9997	0.0252	0.0066
*Brcc3*	0.9778	0.9997	−0.1144	−0.0282
*Cckar*	0.9988	0.9997	−0.0058	−0.0015
*Cd44*	0.9900	0.9997	−0.0764	−0.0127
*Cd74*	0.9584	0.9997	0.1988	0.0529
*Col14a1*	0.9821	0.9997	0.0863	0.0227
*Cops3*	0.9938	0.9997	−0.0417	−0.0079
*Cops5*	0.9967	0.9997	−0.0224	−0.0042
*Cops6*	0.9788	0.9997	−0.1265	−0.0269
*Cox8a*	0.9928	0.9997	0.0541	0.0092
*Ctsf*	0.8714	0.9997	0.6937	0.1642
*Cxcl12*	0.8103	0.9997	1.1162	0.2436
*Cxcl8*	0.7477	0.9997	1.6641	0.3266
*Cxcr1*	0.9895	0.9997	0.0503	0.0133
*Cxcr2*	0.9931	0.9997	−0.0332	−0.0088
*Cxcr4*	0.9865	0.9997	0.0641	0.0171
*Ddt*	0.9830	0.9997	−0.1208	−0.0216
*Ddtl*	0.9864	0.9997	−0.0649	−0.0173
*Dmp1*	0.9995	0.9997	−0.0025	−0.0007
*Dstyk*	0.9135	0.9997	−0.4908	−0.1102
*Dusp14*	0.9551	0.9997	0.2850	0.0571
*Eif3f*	0.9699	0.9997	−0.2166	−0.0382
*Eif3h*	0.9997	0.9997	0.0023	0.0004
*Epcam*	0.9901	0.9997	0.0475	0.0125
*Fpr2*	0.9989	0.9997	0.0050	0.0013
*Hla–Dma*	0.9984	0.9997	−0.0082	−0.0020
*Hla–Dpb1*	0.9938	0.9997	0.0293	0.0078
*Hla–Dqa1*	0.9960	0.9997	0.0190	0.0051
*Igfbp3*	0.8091	0.9997	1.3739	0.2452
*Lamtor5*	0.9795	0.9997	−0.1449	−0.0261
*Lyve1*	0.9951	0.9997	0.0232	0.0062
*Mif*	0.9855	0.9997	−0.1113	−0.0184
*Mpnd*	0.9990	0.9997	0.0048	0.0013
*Ntsr2*	0.9979	0.9997	0.0102	0.0027
*Rgs12*	0.9713	0.9997	0.1623	0.0365
*Rnf139*	0.9994	0.9997	0.0029	0.0008
*St13*	0.9808	0.9997	0.1248	0.0244
*Susd5*	0.9952	0.9997	−0.0235	−0.0061
*Tacstd2*	0.8993	0.9997	0.4954	0.1284
*Uchl1*	0.9456	0.9997	−0.3802	−0.0692
*Vav2*	0.9707	0.9997	0.1509	0.0373

**Table 2:** Results of the Bayesian estimation of temporal regulation analysis for the MIF-related genes in DMD samples from the GSE38417 dataset.

Gene Symbol	ID_REF	Significance-Values
*Ackr3*	1559114_a_at	0.999979
*Adra1a*	211489_at	0.999979
*Amh*	206516_at	0.999981
*Atp13a2*	218608_at	0.99998
*Brcc3*	231913_s_at	0.99998
*Cckar*	211174_s_at	0.99998
*Cd44*	212063_at	0.999968
*Cd74*	1567627_at	0.999978
*Col14a1*	212865_s_at	0.927757
*Cops3*	202078_at	0.999981
*Cops5*	201652_at	0.999974
*Cops6*	201405_s_at	0.99998
*Cox8a*	201119_s_at	0.99998
*Ctsf*	203657_s_at	0.99998
*Cxcl12*	209687_at	0.999979
*Cxcl8*	202859_x_at	0.99998
*Cxcr1*	207094_at	0.999979
*Cxcr2*	207008_at	0.999976
*Cxcr4*	217028_at	0.999979
*Ddt*	202929_s_at	0.99998
*Dmp1*	217067_s_at	0.999981
*Dstyk*	211515_s_at	0.999979
*Eif3f*	200023_s_at	0.99998
*Eif3h*	230570_at	0.999979
*Epcam*	201839_s_at	0.99998
*Fpr2*	210773_s_at	0.999979
*Gtf3a*	201338_x_at	0.99998
*Hbegf*	203821_at	0.999981
*Hla-Dma*	217478_s_at	0.99998
*Hla-Dpb1*	244485_at	0.999975
*Hla-Dqa1*	203290_at	0.999981
*Igfbp3*	210095_s_at	0.999974
*Lamtor5*	202300_at	0.999976
*Lyve1*	219059_s_at	0.999981
*Mif*	217871_s_at	0.999981
*Mpnd*	233651_s_at	0.999981
*Ntsr2*	206899_at	0.99998
*Rgs12*	209639_s_at	0.999965
*Rnf139*	209510_at	0.999981
*St13*	207040_s_at	0.99998
*Susd5*	214954_at	0.99998
*Tacstd2*	202286_s_at	0.999981
*Uchl1*	201387_s_at	0.999979
*Usp14*	201672_s_at	0.999976
*Vav2*	205537_s_at	0.999979

## Supplementary Materials

The following are available online at https://www.mdpi.com/2073-4425/10/11/939/s1, Figure S1: Network of the most significant genes from the meta-analysis and Gene Enrichment for Biological Processes; Table S1: Node-node interactions of the MIF network; Table S2: Most interconnected DEG in the Network of the Meta-analysis.
