# Supplementary material for: Transcriptomic Analysis Reveals Involvement of the Macrophage Migration Inhibitory Factor Gene Network in Duchenne Muscular Dystrophy
Source: Genes (Basel). 2019 Nov 18;10(11):939. doi: 10.3390/genes10110939 (PMC6896047; doi:10.3390/genes10110939)
Supplement: Supplementary file 1 [file genes-10-00939-s001.zip › Suppl Table S2.pdf]

Table S2. Most interconnected DEG in the Network of the Meta-analysis: Cytoscape was interrogated to analyze the STRING Network. This table provides a list of the 50 most interconnected genes in the Network of the Meta-analysis, sorted by degree. Betweenness Centrality, Closeness Centrality and Degree has been considered for centrality parameters of the network. CD44, CXCR4 and CXCL12 are in the top interconnected genes, showing respectively 160, 110 and 104 connections.

Gene	Degree	BetweennessCentrality	ClosenessCentrality
GAPDH	324	0.08614166	0.50774336
FN1	218	0.02825168	0.4642225
VEGFA	195	0.02637694	0.46375347
ACTB	181	0.02985857	0.47028689
APP	167	0.02154225	0.44955926
CD44	160	0.01289183	0.44552293
CASP3	152	0.01892317	0.45762712
PTPRC	149	0.00961433	0.4336325
ITGAM	145	0.00843399	0.43569056
PTEN	142	0.01851706	0.44835165
RHOA	141	0.01729869	0.43965517
STAT1	132	0.01473616	0.43393997
MMP2	122	0.00798742	0.42977528
CCL2	122	0.00688346	0.43353011
MAPK14	119	0.01279147	0.43745533
ITGB2	118	0.00693479	0.41156691
MTOR	114	0.01694883	0.44337117
CXCR4	110	0.00416833	0.4273743
HSPA5	108	0.0157237	0.43404255
VCAM1	107	0.00550744	0.41632653
APOE	105	0.0091125	0.42460685
C3	104	0.00548989	0.40502978
CXCL12	104	0.00353351	0.42236025
COL1A1	101	0.00415999	0.40845384
TPI1	100	0.00960708	0.41604351
ISG15	100	0.00859203	0.40982143
TIMP1	99	0.00241313	0.41670449
PLEK	98	0.00962883	0.40316206
ATP5A1	97	0.00640187	0.40772818
TYROBP	96	0.00449512	0.38138762
CYBB	95	0.00606423	0.42352941
SMAD3	94	0.01077158	0.42549247
TGFB1	93	0.00480146	0.42206897
COL1A2	92	0.00441335	0.4025433
CCL5	92	0.00263859	0.41239892
SPP1	92	0.00264041	0.41576087
CS	91	0.00460332	0.40069838
SDHA	90	0.00553902	0.39982578
CAV1	90	0.00889528	0.43017807
RBX1	89	0.00700816	0.39774697
TLR7	89	0.00328058	0.41388638
COL3A1	88	0.00327177	0.39594565
C3AR1	88	0.00298753	0.39306358
IRF8	88	0.002234	0.38636364
CFL1	87	0.00582694	0.425
FYN	87	0.00576856	0.42206897
RAC2	87	0.00611073	0.41623215
ENO1	86	0.00446053	0.42042592
CANX	85	0.01072667	0.42236025
DCN	85	0.00567503	0.39534884
