# Supplementary material for: Transcriptomic Analysis Reveals Involvement of the Macrophage Migration Inhibitory Factor Gene Network in Duchenne Muscular Dystrophy
Source: Genes (Basel). 2019 Nov 18;10(11):939. doi: 10.3390/genes10110939 (PMC6896047; doi:10.3390/genes10110939)
Supplement: Supplementary file 1 [file genes-10-00939-s001.zip › suppl. Table S1.pdf]

Table S1. Node-node interactions of the MIF network. The GeneMania database was used to construct the MIF network, based on Physical Interaction, Co-expression, Predicted, Co-localization, Pathway, Genetic Interactions and Shared protein domains. Input genes were: *MIF*, *DDT*, *CD74*, *CD44*, *CXCR2*, *CXCR4*, *CXCR7* (a.k.a. *ACKR3*), and *COPS5*. Overall, the network included 48 unique genes with 81 multi-edge node pairs.

Gene 1	Gene 2	Weight	Network group	Network
COPS5	MIF	0.020473	Co-expression	Dobbin-Giordano-2005
EIF3H	RNF139	0.018245	Co-expression	Dobbin-Giordano-2005
VAV2	NTSR2	0.004407	Co-expression	Dobbin-Giordano-2005
COX8A	MIF	0.011951	Co-expression	Dobbin-Giordano-2005
COX8A	COPS5	0.015342	Co-expression	Dobbin-Giordano-2005
HLA-DMA	CD74	0.003402	Co-expression	Dobbin-Giordano-2005
COPS6	COPS5	0.022982	Co-expression	Dobbin-Giordano-2005
EPCAM	TACSTD2	0.010718	Co-expression	Dobbin-Giordano-2005
CXCR1	NTSR2	0.003798	Co-expression	Dobbin-Giordano-2005
HLA-DPB1	CD74	0.004239	Co-expression	Dobbin-Giordano-2005
HLA-DPB1	HLA-DMA	0.004542	Co-expression	Dobbin-Giordano-2005
LAMTOR5	EIF3H	0.017657	Co-expression	Dobbin-Giordano-2005
LYVE1	CTSF	0.012665	Co-expression	Dobbin-Giordano-2005
USP14	COPS5	0.024571	Co-expression	Dobbin-Giordano-2005
USP14	COX8A	0.012683	Co-expression	Dobbin-Giordano-2005
HBEGF	CXCL8	0.018402	Co-expression	Dobbin-Giordano-2005
CCKAR	NTSR2	0.004951	Co-expression	Dobbin-Giordano-2005
CXCR4	CXCR2	0.014793	Co-expression	Wu-Garvey-2007
EIF3H	COPS5	0.003911	Co-expression	Wu-Garvey-2007
EIF3F	CD74	0.008444	Co-expression	Wu-Garvey-2007
EIF3F	CTSF	0.007781	Co-expression	Wu-Garvey-2007
EIF3F	GTF3A	0.005326	Co-expression	Wu-Garvey-2007
CXCR1	CXCR4	0.009696	Co-expression	Wu-Garvey-2007
LAMTOR5	EIF3F	0.004476	Co-expression	Wu-Garvey-2007
COPS3	EIF3H	0.008541	Co-expression	Wu-Garvey-2007
ST13	COPS5	0.003524	Co-expression	Wu-Garvey-2007
CXCR4	CD74	0.0118	Co-expression	Burington-Shaughnessy-2008
GTF3A	EIF3H	0.016951	Co-expression	Burington-Shaughnessy-2008
EPCAM	ACKR3	0.018327	Co-expression	Burington-Shaughnessy-2008
EIF3F	CTSF	0.007426	Co-expression	Burington-Shaughnessy-2008
EIF3F	EIF3H	0.007358	Co-expression	Burington-Shaughnessy-2008
HLA-DPB1	CXCL12	0.007375	Co-expression	Burington-Shaughnessy-2008
HLA-DPB1	HLA-DMA	0.010809	Co-expression	Burington-Shaughnessy-2008
CXCR4	CD74	0.006724	Co-expression	Boldrick-Relman-2002
VAV2	CXCR2	0.020885	Co-expression	Boldrick-Relman-2002

HLA-DMA	CXCR4	0.009169	Co-expression	Boldrick-Relman-2002
HLA-DPB1	ACKR3	0.00545	Co-expression	Boldrick-Relman-2002
HLA-DPB1	CD74	0.003015	Co-expression	Boldrick-Relman-2002
HLA-DPB1	HLA-DMA	0.004373	Co-expression	Boldrick-Relman-2002
ST13	COPS5	0.012436	Co-expression	Boldrick-Relman-2002
CD74	DDT	0.003213	Genetic Interactions	Lin-Smith-2010
CXCR2	DDT	0.001281	Genetic Interactions	Lin-Smith-2010
CXCR4	CXCR2	0.000933	Genetic Interactions	Lin-Smith-2010
CXCR4	CD44	0.000694	Genetic Interactions	Lin-Smith-2010
CXCL12	CD44	0.000523	Genetic Interactions	Lin-Smith-2010
DMP1	ACKR3	0.001393	Genetic Interactions	Lin-Smith-2010
DMP1	MIF	0.002854	Genetic Interactions	Lin-Smith-2010
DMP1	CD44	0.000873	Genetic Interactions	Lin-Smith-2010
UCHL1	CD44	0.000693	Genetic Interactions	Lin-Smith-2010
UCHL1	CXCL12	0.000342	Genetic Interactions	Lin-Smith-2010
COL14A1	DMP1	0.001237	Genetic Interactions	Lin-Smith-2010
RGS12	BNIPL	0.001357	Genetic Interactions	Lin-Smith-2010
VAV2	MIF	0.002372	Genetic Interactions	Lin-Smith-2010
VAV2	CD74	0.00245	Genetic Interactions	Lin-Smith-2010
CXCL8	CXCR4	0.00078	Genetic Interactions	Lin-Smith-2010
CXCL8	CXCL12	0.000588	Genetic Interactions	Lin-Smith-2010
COX8A	VAV2	0.001082	Genetic Interactions	Lin-Smith-2010
TACSTD2	CXCL12	0.000937	Genetic Interactions	Lin-Smith-2010
ATP13A2	CXCR4	0.003035	Genetic Interactions	Lin-Smith-2010
EPCAM	CXCR4	0.000715	Genetic Interactions	Lin-Smith-2010
GRK3	CD74	0.002588	Genetic Interactions	Lin-Smith-2010
GRK3	UCHL1	0.000501	Genetic Interactions	Lin-Smith-2010
GRK3	RGS12	0.00072	Genetic Interactions	Lin-Smith-2010
IGFBP3	EPCAM	0.001338	Genetic Interactions	Lin-Smith-2010
IGFBP3	EIF3F	0.003026	Genetic Interactions	Lin-Smith-2010
ADRA1A	CXCL12	0.00034	Genetic Interactions	Lin-Smith-2010
LAMTOR5	CXCR4	0.00125	Genetic Interactions	Lin-Smith-2010
DSTYK	MIF	0.002857	Genetic Interactions	Lin-Smith-2010
DSTYK	EIF3H	0.001342	Genetic Interactions	Lin-Smith-2010
DSTYK	CXCL8	0.000983	Genetic Interactions	Lin-Smith-2010
DSTYK	GRK3	0.000632	Genetic Interactions	Lin-Smith-2010
LYVE1	GRK3	0.001104	Genetic Interactions	Lin-Smith-2010
LYVE1	DSTYK	0.001259	Genetic Interactions	Lin-Smith-2010
COPS3	CD44	0.00138	Genetic Interactions	Lin-Smith-2010
COPS3	DSTYK	0.001138	Genetic Interactions	Lin-Smith-2010
HBEGF	BNIPL	0.002979	Genetic Interactions	Lin-Smith-2010
ST13	UCHL1	0.001158	Genetic Interactions	Lin-Smith-2010
CCKAR	NTSR2	0.003091	Genetic Interactions	Lin-Smith-2010

CCKAR	ST13	0.001631	Genetic Interactions	Lin-Smith-2010
CD74	MIF	0.720807	Physical Interactions	IREF-BIND
CXCL12	CXCR4	0.940235	Physical Interactions	IREF-BIND
BNIPL	MIF	0.230385	Physical Interactions	IREF-BIND
CXCR1	CXCL8	0.521285	Physical Interactions	IREF-BIND
CD74	MIF	0.440914	Physical Interactions	BIOGRID-SMALL-SCALE-STUDIES
COPS5	MIF	0.031229	Physical Interactions	BIOGRID-SMALL-SCALE-STUDIES
CXCL12	CXCR4	0.150551	Physical Interactions	BIOGRID-SMALL-SCALE-STUDIES
BNIPL	MIF	0.225817	Physical Interactions	BIOGRID-SMALL-SCALE-STUDIES
UCHL1	COPS5	0.011161	Physical Interactions	BIOGRID-SMALL-SCALE-STUDIES
COL14A1	CD44	0.213284	Physical Interactions	BIOGRID-SMALL-SCALE-STUDIES
RGS12	CXCR2	0.272348	Physical Interactions	BIOGRID-SMALL-SCALE-STUDIES
VAV2	CD44	0.064289	Physical Interactions	BIOGRID-SMALL-SCALE-STUDIES
CXCL8	CXCR2	0.256641	Physical Interactions	BIOGRID-SMALL-SCALE-STUDIES
GTF3A	CXCR2	0.237576	Physical Interactions	BIOGRID-SMALL-SCALE-STUDIES
ATP13A2	ACKR3	0.200508	Physical Interactions	BIOGRID-SMALL-SCALE-STUDIES
ATP13A2	CXCR4	0.01659	Physical Interactions	BIOGRID-SMALL-SCALE-STUDIES
COPS6	COPS5	0.012099	Physical Interactions	BIOGRID-SMALL-SCALE-STUDIES
GRK3	CXCR4	0.1637	Physical Interactions	BIOGRID-SMALL-SCALE-STUDIES
IGFBP3	CD44	0.04173	Physical Interactions	BIOGRID-SMALL-SCALE-STUDIES
LAMTOR5	CD74	0.16512	Physical Interactions	BIOGRID-SMALL-SCALE-STUDIES
COPS3	COPS5	0.010025	Physical Interactions	BIOGRID-SMALL-SCALE-STUDIES
COPS3	COPS6	0.028202	Physical Interactions	BIOGRID-SMALL-SCALE-STUDIES
USP14	CXCR4	0.072892	Physical Interactions	BIOGRID-SMALL-SCALE-STUDIES
HBEGF	CD44	0.079285	Physical Interactions	BIOGRID-SMALL-SCALE-STUDIES
AMH	COPS5	0.209435	Physical Interactions	BIOGRID-SMALL-SCALE-STUDIES
ST13	CXCR2	0.125709	Physical Interactions	BIOGRID-SMALL-SCALE-STUDIES
ST13	CXCR4	0.06617	Physical Interactions	BIOGRID-SMALL-SCALE-STUDIES

BRCC3	COPS5	0.019148	Physical Interactions	BIOGRID-SMALL-SCALE-STUDIES
CD74	MIF	0.308792	Physical Interactions	IREF-BIOGRID
COPS5	MIF	0.004278	Physical Interactions	IREF-BIOGRID
CXCL12	CXCR4	0.154938	Physical Interactions	IREF-BIOGRID
BNIPL	MIF	0.08035	Physical Interactions	IREF-BIOGRID
UCHL1	COPS5	0.002302	Physical Interactions	IREF-BIOGRID
COL14A1	CD44	0.180092	Physical Interactions	IREF-BIOGRID
RGS12	CXCR2	0.293855	Physical Interactions	IREF-BIOGRID
VAV2	CD44	0.018483	Physical Interactions	IREF-BIOGRID
CXCL8	CXCR2	0.143705	Physical Interactions	IREF-BIOGRID
GTF3A	CXCR2	0.27779	Physical Interactions	IREF-BIOGRID
ATP13A2	ACKR3	0.193558	Physical Interactions	IREF-BIOGRID
ATP13A2	CXCR4	0.020981	Physical Interactions	IREF-BIOGRID
COPS6	COPS5	0.000519	Physical Interactions	IREF-BIOGRID
EIF3F	EIF3H	0.016239	Physical Interactions	IREF-BIOGRID
GRK3	CXCR4	0.164392	Physical Interactions	IREF-BIOGRID
IGFBP3	CD44	0.044728	Physical Interactions	IREF-BIOGRID
DSTYK	DDT	0.428263	Physical Interactions	IREF-BIOGRID
COPS3	COPS5	0.001956	Physical Interactions	IREF-BIOGRID
COPS3	COPS6	0.003959	Physical Interactions	IREF-BIOGRID
USP14	CXCR4	0.033472	Physical Interactions	IREF-BIOGRID
USP14	COPS5	0.002311	Physical Interactions	IREF-BIOGRID
HBEGF	CD44	0.087266	Physical Interactions	IREF-BIOGRID
AMH	COPS5	0.012252	Physical Interactions	IREF-BIOGRID
ST13	CXCR2	0.084032	Physical Interactions	IREF-BIOGRID
ST13	CXCR4	0.043125	Physical Interactions	IREF-BIOGRID
ST13	VAV2	0.019751	Physical Interactions	IREF-BIOGRID
BRCC3	COPS5	0.002512	Physical Interactions	IREF-BIOGRID
CXCR4	CD74	0.115724	Physical Interactions	IREF-INTACT
COPS5	MIF	0.023423	Physical Interactions	IREF-INTACT
UCHL1	COPS5	0.029864	Physical Interactions	IREF-INTACT
CXCL8	CXCR2	0.438321	Physical Interactions	IREF-INTACT
ATP13A2	ACKR3	0.073324	Physical Interactions	IREF-INTACT
ATP13A2	CXCR4	0.025381	Physical Interactions	IREF-INTACT
HLA-DQA1	CD74	0.135741	Physical Interactions	IREF-INTACT
COPS6	MIF	0.018174	Physical Interactions	IREF-INTACT
DSTYK	DDT	0.270535	Physical Interactions	IREF-INTACT
DSTYK	MIF	0.062623	Physical Interactions	IREF-INTACT
USP14	COPS5	0.09164	Physical Interactions	IREF-INTACT
ST13	VAV2	0.020156	Physical Interactions	IREF-INTACT
MIF	DDT	0.645584	Predicted	Wu-Stein-2010
CD74	MIF	0.106819	Predicted	Wu-Stein-2010
CXCR4	CXCR2	0.034322	Predicted	Wu-Stein-2010
CTSF	CD74	0.469814	Predicted	Wu-Stein-2010

NTSR2	CXCR2	0.500937	Predicted	Wu-Stein-2010
DMP1	CD44	0.364803	Predicted	Wu-Stein-2010
UCHL1	MIF	0.308777	Predicted	Wu-Stein-2010
RNF139	COPS5	0.349848	Predicted	Wu-Stein-2010
COL14A1	CD44	0.103879	Predicted	Wu-Stein-2010
EIF3H	COPS5	0.169901	Predicted	Wu-Stein-2010
VAV2	CD44	0.226558	Predicted	Wu-Stein-2010
COX8A	MIF	0.161149	Predicted	Wu-Stein-2010
HLA-DMA	CD74	0.19275	Predicted	Wu-Stein-2010
HLA-DQA1	CD74	0.107949	Predicted	Wu-Stein-2010
HLA-DQA1	CXCR4	0.053159	Predicted	Wu-Stein-2010
COPS6	COPS5	0.120707	Predicted	Wu-Stein-2010
EIF3F	COPS5	0.123082	Predicted	Wu-Stein-2010
FPR2	CXCR2	0.142713	Predicted	Wu-Stein-2010
CXCR1	CXCR2	0.084536	Predicted	Wu-Stein-2010
GRK3	CXCR4	0.040105	Predicted	Wu-Stein-2010
HLA-DPB1	CD74	0.127471	Predicted	Wu-Stein-2010
IGFBP3	CD44	0.061121	Predicted	Wu-Stein-2010
ADRA1A	CXCR2	0.1292	Predicted	Wu-Stein-2010
ADRA1A	CXCR1	0.137639	Predicted	Wu-Stein-2010
COPS3	COPS5	0.197019	Predicted	Wu-Stein-2010
USP14	COPS5	0.109278	Predicted	Wu-Stein-2010
HBEGF	CD44	0.065261	Predicted	Wu-Stein-2010
CCKAR	CXCR2	0.112857	Predicted	Wu-Stein-2010
MIF	DDT	0.5	Shared protein domains	PFAM
CXCR2	ACKR3	0.003514	Shared protein domains	PFAM
CXCR4	ACKR3	0.003658	Shared protein domains	PFAM
CXCR4	CXCR2	0.003627	Shared protein domains	PFAM
DDTL	DDT	0.5	Shared protein domains	PFAM
DDTL	MIF	0.5	Shared protein domains	PFAM
NTSR2	ACKR3	0.003544	Shared protein domains	PFAM
NTSR2	CXCR2	0.003514	Shared protein domains	PFAM
NTSR2	CXCR4	0.003658	Shared protein domains	PFAM
EIF3H	COPS5	0.088474	Shared protein domains	PFAM
CXCL8	CXCL12	0.022222	Shared protein domains	PFAM

TACSTD2	CD74	0.096679	Shared protein domains	PFAM
HLA-DQA1	HLA-DMA	0.078386	Shared protein domains	PFAM
COPS6	COPS5	0.066196	Shared protein domains	PFAM
COPS6	EIF3H	0.066196	Shared protein domains	PFAM
EPCAM	CD74	0.096679	Shared protein domains	PFAM
EPCAM	TACSTD2	0.096679	Shared protein domains	PFAM
EIF3F	COPS5	0.066196	Shared protein domains	PFAM
EIF3F	EIF3H	0.066196	Shared protein domains	PFAM
EIF3F	COPS6	0.111219	Shared protein domains	PFAM
FPR2	ACKR3	0.003544	Shared protein domains	PFAM
FPR2	CXCR2	0.003514	Shared protein domains	PFAM
FPR2	CXCR4	0.003658	Shared protein domains	PFAM
FPR2	NTSR2	0.003544	Shared protein domains	PFAM
CXCR1	ACKR3	0.003544	Shared protein domains	PFAM
CXCR1	CXCR2	0.003514	Shared protein domains	PFAM
CXCR1	CXCR4	0.003658	Shared protein domains	PFAM
CXCR1	NTSR2	0.003544	Shared protein domains	PFAM
CXCR1	FPR2	0.003544	Shared protein domains	PFAM
GRK3	RGS12	0.011036	Shared protein domains	PFAM
HLA-DPB1	HLA-DMA	0.017238	Shared protein domains	PFAM
HLA-DPB1	HLA-DQA1	0.017238	Shared protein domains	PFAM
IGFBP3	CD74	0.055422	Shared protein domains	PFAM
IGFBP3	TACSTD2	0.055422	Shared protein domains	PFAM
IGFBP3	EPCAM	0.055422	Shared protein domains	PFAM
ADRA1A	ACKR3	0.003544	Shared protein domains	PFAM
ADRA1A	CXCR2	0.003514	Shared protein domains	PFAM

ADRA1A	CXCR4	0.003658	Shared protein domains	PFAM
ADRA1A	NTSR2	0.003544	Shared protein domains	PFAM
ADRA1A	FPR2	0.003544	Shared protein domains	PFAM
ADRA1A	CXCR1	0.003544	Shared protein domains	PFAM
MPND	COPS5	0.088473	Shared protein domains	PFAM
MPND	EIF3H	0.088473	Shared protein domains	PFAM
MPND	COPS6	0.066196	Shared protein domains	PFAM
MPND	EIF3F	0.066196	Shared protein domains	PFAM
LYVE1	CD44	0.12245	Shared protein domains	PFAM
SUSD5	CD44	0.12245	Shared protein domains	PFAM
SUSD5	LYVE1	0.12245	Shared protein domains	PFAM
BRCC3	COPS5	0.088474	Shared protein domains	PFAM
BRCC3	EIF3H	0.088474	Shared protein domains	PFAM
BRCC3	COPS6	0.066196	Shared protein domains	PFAM
BRCC3	EIF3F	0.066196	Shared protein domains	PFAM
BRCC3	MPND	0.088473	Shared protein domains	PFAM
CCKAR	ACKR3	0.003658	Shared protein domains	PFAM
CCKAR	CXCR2	0.003627	Shared protein domains	PFAM
CCKAR	NTSR2	0.003658	Shared protein domains	PFAM
CCKAR	FPR2	0.003658	Shared protein domains	PFAM
CCKAR	CXCR1	0.003658	Shared protein domains	PFAM
CCKAR	ADRA1A	0.003658	Shared protein domains	PFAM
MIF	DDT	0.5	Shared protein domains	INTERPRO
DDTL	DDT	0.5	Shared protein domains	INTERPRO
DDTL	MIF	0.5	Shared protein domains	INTERPRO
EIF3H	COPS5	0.093544	Shared protein domains	INTERPRO

CXCL8	CXCL12	0.01755	Shared protein domains	INTERPRO
TACSTD2	CD74	0.12571	Shared protein domains	INTERPRO
HLA-DQA1	HLA-DMA	0.041896	Shared protein domains	INTERPRO
COPS6	COPS5	0.069395	Shared protein domains	INTERPRO
COPS6	EIF3H	0.069395	Shared protein domains	INTERPRO
EPCAM	CD74	0.12571	Shared protein domains	INTERPRO
EPCAM	TACSTD2	0.125711	Shared protein domains	INTERPRO
EIF3F	COPS5	0.069395	Shared protein domains	INTERPRO
EIF3F	EIF3H	0.069395	Shared protein domains	INTERPRO
EIF3F	COPS6	0.115446	Shared protein domains	INTERPRO
FPR2	ACKR3	0.008256	Shared protein domains	INTERPRO
FPR2	CXCR2	0.007546	Shared protein domains	INTERPRO
FPR2	CXCR4	0.004277	Shared protein domains	INTERPRO
FPR2	NTSR2	0.00756	Shared protein domains	INTERPRO
CXCR1	CXCR2	0.133662	Shared protein domains	INTERPRO
CXCR1	FPR2	0.007546	Shared protein domains	INTERPRO
GRK3	RGS12	0.006683	Shared protein domains	INTERPRO
HLA-DPB1	HLA-DMA	0.020753	Shared protein domains	INTERPRO
HLA-DPB1	HLA-DQA1	0.020753	Shared protein domains	INTERPRO
IGFBP3	CD74	0.042247	Shared protein domains	INTERPRO
IGFBP3	TACSTD2	0.042247	Shared protein domains	INTERPRO
IGFBP3	EPCAM	0.042247	Shared protein domains	INTERPRO
ADRA1A	FPR2	0.00536	Shared protein domains	INTERPRO
MPND	COPS5	0.093544	Shared protein domains	INTERPRO
MPND	EIF3H	0.093544	Shared protein domains	INTERPRO
MPND	COPS6	0.069395	Shared protein domains	INTERPRO

MPND	EIF3F	0.069395	Shared protein domains	INTERPRO
LYVE1	CD44	0.054116	Shared protein domains	INTERPRO
SUSD5	CD44	0.043092	Shared protein domains	INTERPRO
SUSD5	LYVE1	0.043092	Shared protein domains	INTERPRO
BRCC3	COPS5	0.093544	Shared protein domains	INTERPRO
BRCC3	EIF3H	0.093544	Shared protein domains	INTERPRO
BRCC3	COPS6	0.069395	Shared protein domains	INTERPRO
BRCC3	EIF3F	0.069395	Shared protein domains	INTERPRO
BRCC3	MPND	0.093544	Shared protein domains	INTERPRO
CCKAR	FPR2	0.007546	Shared protein domains	INTERPRO
