# Supplementary material for: Transcriptomic Analysis Reveals Involvement of the Macrophage Migration Inhibitory Factor Gene Network in Duchenne Muscular Dystrophy
Source: Genes (Basel). 2019 Nov 18;10(11):939. doi: 10.3390/genes10110939 (PMC6896047; doi:10.3390/genes10110939)
Supplement: Supplementary file 1 [file genes-10-00939-s001.zip › Supplementary Figure Legend.pdf]

Suppl. Figure Legend

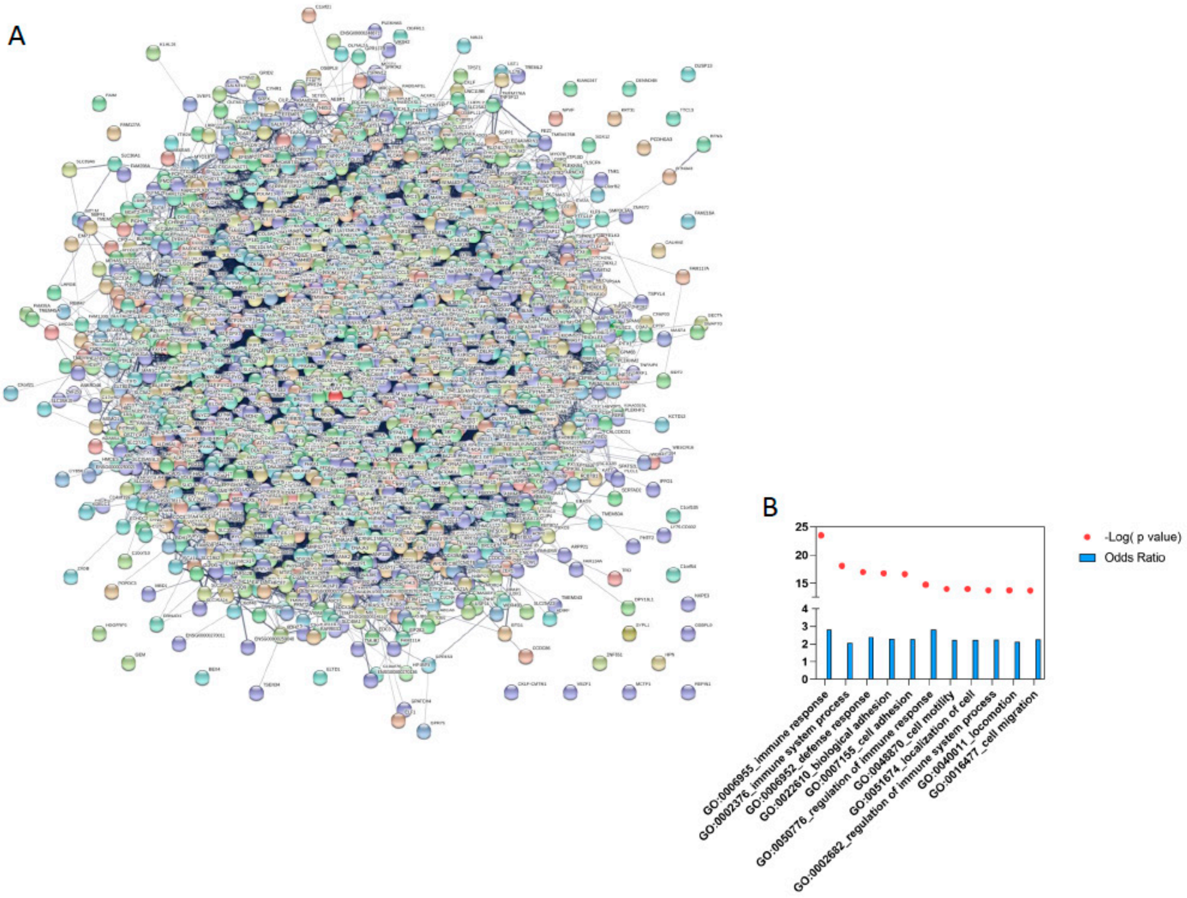

Figure 1. Network of the most significant genes from the meta-analysis (**A**) Gene network of the 2000 top genes altered in the Meta-analysis is plotted using STRING. The edges connecting the nodes represent the interactions between genes, in terms of co-expression (black), text mining (light green), protein homology (cyan), association in curated database (light blue), and high-throughput experiments (purple). Empty nodes represent proteins of uncharacterized 3D structure, while filled nodes represent proteins with known or predicted tertiary structure. Gene Enrichment Biological Process of the Meta-Analysis **(B)** Enrichment analysis is performed with ImaGEO webtool. Red dots represent the significance of the annotations as Log (p-value), while blue histograms show the Odds Ratio for each category tested. It is shown a significant up-regulation of the immune response (p-value 3.2e-24) and of the immune system (*p*-value 8.2e-19).
